# A New Phylogeographic Pattern of Endemic *Bufo bankorensis* in Taiwan Island Is Attributed to the Genetic Variation of Populations

**DOI:** 10.1371/journal.pone.0098029

**Published:** 2014-05-22

**Authors:** Teng-Lang Yu, Hung-Du Lin, Ching-Feng Weng

**Affiliations:** 1 Department of Life Science and Institute of Biotechnology, National Dong Hwa University, Hualien, Taiwan; 2 The Affiliated School of National Tainan First Senior High School, Tainan, Taiwan; Institute of Biochemistry and Biology, Germany

## Abstract

**Aim:**

To comprehend the phylogeographic patterns of genetic variation in anurans at Taiwan Island, this study attempted to examine (1) the existence of various geological barriers (Central Mountain Ranges, CMRs); and (2) the genetic variation of *Bufo bankorensis* using mtDNA sequences among populations located in different regions of Taiwan, characterized by different climates and existing under extreme conditions when compared available sequences of related species *B. gargarizans* of mainland China.

**Methodology/Principal Findings:**

Phylogenetic analyses of the dataset with mitochondrial DNA (mtDNA) D-loop gene (348 bp) recovered a close relationship between *B. bankorensis* and *B. gargarizans*, identified three distinct lineages. Furthermore, the network of mtDNA D-loop gene (564 bp) amplified (279 individuals, 27 localities) from Taiwan Island indicated three divergent clades within *B. bankorensis* (Clade W, E and S), corresponding to the geography, thereby verifying the importance of the CMRs and Kaoping River drainage as major biogeographic barriers. Mismatch distribution analysis, neutrality tests and Bayesian skyline plots revealed that a significant population expansion occurred for the total population and Clade W, with horizons dated to approximately 0.08 and 0.07 Mya, respectively. These results suggest that the population expansion of Taiwan Island species *B. bankorensis* might have resulted from the release of available habitat in post-glacial periods, the genetic variation on mtDNA showing habitat selection, subsequent population dispersal, and co-distribution among clades.

**Conclusions:**

The multiple origins (different clades) of *B. bankorensis* mtDNA sequences were first evident in this study. The divergent genetic clades found within *B. bankorensis* could be independent colonization by previously diverged lineages; inferring *B. bankorensis* originated from *B. gargarizans* of mainland China, then dispersal followed by isolation within Taiwan Island. Highly divergent clades between W and E of *B. bankorensis*, implies that the CMRs serve as a genetic barrier and separated the whole island into the western and eastern phylogroups.

## Introduction

**Figure 1:**
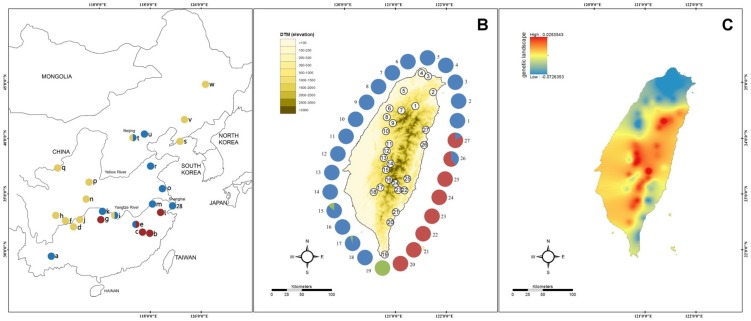
Genetic and topographic maps in Taiwan and mainland China. (A) *B. gargarizans* in various regions (23, a-w) of mainland China retrieved from NCBI GenBank correspond to the locations and sampling site (28, Shanghai). (B) The topographic landscape of Taiwan and *B. bankorensis* sampling localities (27, 1–27) given in Table 1. (C) Genetic landscape deduced from *B. bankorensis* D-loop sequences as compared to the topographic landscape of Taiwan. The colors are used in the pie charts corresponding to the proportion of haplotype frequencies of clade or lineage given in Figure 2. Yellow: Lineage I; Blue: Lineage II; Red: Lineage III; Green: Clade S.

Biogeography focuses on the study of the distribution of organisms and populations in distinct geographic space through geological time. Organisms and populations commonly vary in a highly regular fashion with geographic gradients of latitude and altitude, and population differentiation is the result of vicariance and dispersal [1], [2], [3]. Phylogeographic studies examine the present-day distributions of species which can be determined by exploring the relationship between their population genealogy and geographical distribution via the analysis of molecular characteristics, e.g., their demographic history, spatial distribution, population differentiation, and genetic diversity [2], [4], [5]. The effects of geological events on the diverse biota of islands have become the subject of increasing numbers of phylogeographic studies focused on the genetic patterns and processes involved in colonization and speciation [6]. Taiwan Island, a small island (35,830 km^2^) located off the southeastern coast of mainland China and separated from China by the shallow Taiwan Strait, emerged above sea level as the result of a series of collisions between the Philippine Sea Plate and the Eurasian Continental Plate approximately 5 million years ago (Mya) in the Pliocene [7], [8], [9] (Figure 1). The continuous glacial-interglacial cycles of the Pleistocene had an intensive effect on the population distributions of living organisms. At the time of the glacial maxima, temperate regions were largely covered by ice, forcing species to migrate south toward refugia in the Northern Hemisphere [10], while most tropical and subtropical zones displayed cooler and drier climates than today, leading to the displacement of moist forests by xerophytic vegetation [10], [11]. Glaciations might also have provided opportunities for species to migrate between different altitudes. Quaternary climate oscillations often occurred at an extreme speed and resulted in repeated severe environmental changes, causing massive range shifts among biota [10], [12], [13]. The ongoing arc-continent collision resulted in the dramatic rise of the Central Mountain Ranges (CMRs) are aligning from the north of the Taiwan island to the south (Figure 1B), which reached their current elevations approximately 1 to 2.5 Mya [14], [15]. The CMRs, which include more than 260 peaks above 3,000 m, are a major biological barrier along the north-south axis and provide the niches leading to the genetic diversification of endemic organisms [16], such as freshwater fish [17], [18], [19], frogs [20], and squirrels [21]. Two additional topographic barriers, the Miaoli Plateau and Formosa Bank, divided western Taiwan into three phylogeographical areas [18], [20]. This scenario is supported by the phylogenetic signatures obtained for a number of taxa, including frogs such as *Buergeria robusta* [20] and *Sylvirana latouchii* [22], and freshwater fishes such as *Acrossocheilus paradoxus* [23], *Varicorhinus barbatulus* [18], *Hemimyzon formosanus* [24], and *Formosania lacustre* [25]. Due to differences in the dispersal ability of species and ecological constraints, various phylogeographic patterns should be observed among different scenarios. Whether these patterns can be applied to other amphibians particularly toad remains to be verified.

The toad species *Bufo bankorensis* (Barbour, 1908) is widely dispersed across all of Taiwan Island at altitudes between 0 and 3000 m [26]. *B. bankorensis* belongs to the family Bufonidae, which is one of the most species rich (with more than 350 species) and widely distributed amphibian families. According to previous studies, the classification of *B. bankorensis* has been generally debated. Some authors have recognized it as a distinct species [27], [28], [29], while others have synonymized it with *B. gargaizans*, which is distributed throughout mainland China [30]. *B. gargarizans* and *B. bankorensis* were classified as two subspecies present in Taiwan by Kawamura [31], [32] and Nishioka [33] based on the reproductive isolation mechanisms elucidated by crossing experiments, while *B. bankorensis* was reclassified as a distinct endemic species of Taiwan Island by analysis of numerous morphometric characters [28]. Moreover, *B. bankorensis* has been included in the *B. gargarizans* species complex (e.g. [34], [35], [36], [37]). Recent molecular phylogenetic studies in Asian *Bufo* mtDNA [34], [38] demonstrated that *B. bankorensis* should be synonymized with *B. gargarizans* as it constitutes one lineage of this species. However, these results are based on a limited sampling of individuals and localities in Taiwan Island. Many studies have been focused on the structure of genetic variability on the family Bufonidae at different regions to clarify the evolutionary relationships and biogeography patterns e.g. *Bufo bufo* in the Far East and Europe [39], *Bufo woodhousei* [40], *Bufo fowleri* at the Lake Erie basin [41], and *Bufo punctatus* in western North America [42]. Recently, one report revealed that various ecological conditions in a relatively small area have little effect on genetic variation in the green toad *Pseudepidalea viridis* throughout Israel [43]. Toads have often demonstrated both strong site fidelity for breeding ponds and low dispersal ability [44], [45]. As a result of restricted dispersal capabilities, the varying levels of physiological fitness observed under different environments tend to promote differentiation and speciation [46]. On the other hand, frogs are an excellent taxon for conducting studies of phylogeographic diversification [47], [48], [49], [50] and frog species often show the increased population-genetic structure relative to species without such dispersal limitations [51], [52]. Moreover, frogs are highly sensitive to climatic fluctuations due to their complex amphibian life histories, permeable skin and exposed eggs, and past climatic records from eastern Asia indicate the potential to promote the population divergence and speciation processes [53]. Two major hypotheses have been proposed to explain the genetic variation pattern happened on the island. One is in situ geographical differentiation, where variation has been generated due to discontinue of gene flow by physical barriers within-island. The other is the immigration from the mainland or nearby islands, in which historical climatic oscillations combined with species or population multi-invasion by land bridge, then dispersal followed by isolation, have changed the demographic and distributional patterns of species. To examine the possibility of multiple invasions and isolation hypothesis to the phylogeographic patterns of *B. bankorensis*, this study attempted to examine the genetic variation of *B. bankorensis* using mtDNA sequences among populations located in different regions of Taiwan, characterized by different climates and existing under extreme conditions when compared available mtDNA sequences of related species *B. gargarizan* from mainland China. This information on the genetic variation and diversity among different localities in Taiwan, where the climate and ecological conditions vary dramatically from western to eastern, is integrated to explore the unsolved biogeographical pattern of the wide distribution of Taiwanese *B. bankorensis*.

## Materials and Methods

### Sampling

Some animal collections in the area of national parks were permitted by the Headquarters of Taroko National Park (permission numbers 0990010921, 0990011963, 1000011365, and 1010011630), Yangmingshan National Park (permission number 20110118) and Yushan National Park (permission number 1010210), respectively. *B. bankorensis* is not an endangered or a protected species in Taiwan, the other locations that no specific permissions are required. All experimental protocols were approved by the Institutional Animal Care and Use Committee of Dong Hwa University, Taiwan, and conformed to the guidelines set forth by the International Association for the Study of Pain [54]. From 2010 to 2012, different populations of *B. bankorensis* were sampled around Taiwan Island, with a total 279 individuals being collected from 27 localities and 4 individuals of *B. gargarizans* from Shanghai (mainland China) as related species were also collected and analyzed (Table 1; Figure 1). Samples were obtained from either tadpole fin clips or adult toe clips from live specimens, followed by release of the sampled individuals at their capture localities. The tissue samples were preserved in 95% ethanol and then transferred to a −20°C freezer.

**Table 1:** List of sampling locations and the latitudes and longitudes, sample sizes, haplotype numbers, haplotype diversities (*h*), nucleotide diversities (π), and haplotypes of mtDNA D-loop region sequences for each location.

	Locations*(Abbreviation)	Latitude and longitude	Sample size	Haplotype numbers	Haplotype diversity (*h*)	Nucleotide diversity (π)	mtDNA Haplotype
	**Total**		**279**	**76**	**0.973**	**0.026**	
	**West region**						
1	Cilan (CL)	N24.58°, E121.38°	7	7	1.000	0.008	6,10,17,20,21,22,23
2	Wufongci (WF)	N24.83°, E121.74°	12	4	0.712	0.006	3,4,8,28
3	Yangmingshan Zhuzihu (YZ)	N25.16°, E121.54°	11	4	0.673	0.008	8,44,45,46
4	Yangmingshan Erziping (YE)	N25.18°, E121.52°	18	4	0.654	0.006	8,45,46,47
5	Longtan (LT)	N24.85°, E121.16°	9	2	0.556	0.002	1,22
6	Gongguan (GG)	N24.53°, E120.88°	5	1	0.000	0.000	39
7	Guanwu (GG)	N24.50°, E121.11°	6	6	1.000	0.006	6,7,8,9,10,11
8	Dahu (DH)	N24.38°, E120.85°	5	1	0.000	0.000	50
9	Heping (HP)	N24.28°, E120.94°	10	4	0.733	0.003	6,22,48,49
10	Tungshih (TS)	N24.17°, E120.83°	10	1	0.000	0.000	6
11	Lienhuachih (LC)	N23.89°, E120.89°	10	1	0.000	0.000	16
12	Shueili (SL)	N23.81°, E120.85°	6	6	1.000	0.006	6,16,17,18,33,34
13	Xitou (XT)	N23.67°, E120.79°	7	5	0.905	0.006	6,27,28,29,30
14	Dongpu (DP)	N23.55°, E120.91°	18	12	0.935	0.005	6,12,13,14,15,24,27,35,36,37,38,39
15	Zihjhong (ZJ)	N23.48°, E120.82°	15	10	0.933	0.016	6,15,19,24,25,26,27,28,52,54
16	Jhongjhihguan (JG)	N23.28°, E120.90°	4	4	1.000	0.004	28,31,32,33
17	Gaozhong (GZ)	N23.13°, E120.71°	19	8	0.842	0.010	24,25,32,33,41,42,43,54
18	Jiasian (JS)	N23.08°, E120.58°	12	3	0.621	0.003	33,40,41
19	Kenting (KT)	N21.94°, E120.80°	4	3	0.833	0.003	51,53,55
	**East region**						
20	Jinlun (JL)	N22.52°, E120.93°	7	2	0.286	0.001	70,71
21	Jhihben (JB)	N22.69°, E121.01°	9	1	0.000	0.000	76
22	Chulai (UL)	N23.11°, E121.17°	6	4	0.867	0.003	57,62,67,68
23	Xiama (XM)	N23.15°, E121.06°	5	2	0.600	0.001	56,66
24	Motian (MT)	N23.19°, E121.02°	25	7	0.787	0.003	56,57,62,63,64,65,66
25	Shanfong (SF)	N23.32°, E121.23°	14	7	0.879	0.004	56,57,58,59,60,61,62
26	Coastal Mountain (CM)	N23.98°, E121.58°	5	4	0.900	0.032	2,22,57,69
27	Shakadang (SK)	N24.15°, E121.62°	20	7	0.642	0.014	5,22,33,72,73,74,75
	**mainland China**		**4**	**4**	**1.000**	**0.006**	
28	Shanghai (SH)	N31.19°, E121.32°	4	4	1.000	0.006	77,78,79,80

*locations 3, 4, 14, 16, 25, and 27 belong to national park areas with permission for sampling. Other locations are without permission.

### Molecular Analyses

Total DNAs were extracted using genomic QuickExtract DNA Extraction Solution (DNA Extraction Kit, Epicentre Technologies, Madison, WI, USA) following the manufacturer's manual. A section of the mtDNA control region (D-loop) was sequenced following PCR amplification with the following primers: forward (Bu-con-15971F: GAG CCT TCC CTT GGT TTA AGA GTA) and reverse (Bu-con-16582R: CCA GGT TAA GGT CTT TAA GGT ACC AG) (designed in this study). PCR amplifications were carried out in a 25 µl reaction volume through 35 cycles of denaturing at 94°C for 30–45 sec; annealing at 56°C for 30–45 sec, and 72°C extension for 30–45 sec. The reaction products were mixed with Novel juice (GeneTex, San Antonio, TX, USA) and separated via gel electrophoresis in 1.5% agarose gels. The gels were stained with ethidium bromide, and the desired DNA band was excised and eluted using an agarose gel purification kit (QIAGEN, Valencia, CA, USA). The PCR products were then subjected to cycle sequencing reactions conducted by Genomics Biotec Co., Ltd. (Taiwan) using an ABI PRISM 3730XL sequencer with the BigDye Terminator kit (Applied Biosystems). All sequences have been deposited in GenBank under the following inclusive accession numbers: KF692208-KF692283.

### Data Analyses

#### Genetic Diversity, Phylogenetic, and Phylogeographic Analysis

Partial sequences of the mtDNA D-loop gene were aligned using the program Clustal X v1.81 [55] and optimized manually. Firstly, 80 mtDNA D-loop (348 bp) haplotypes of this study with 38 sequences of *B. gargarizans* in various regions of mainland China were retrieved from NCBI GenBank (Figure 1A, Table 2). One closely related species *Bufo tibetanus* as an outgroup was also obtained from GenBank (UX878885.1). The haplotype genealogy of *B. bankorensis* was separately reconstructed by maximum likelihood (ML) tree, neighbor-joining (NJ) tree and Bayesian analysis using PhyML 3.0 [56], MEGA v5.0 [57] and MrBayes v3.1.2 [58]. Neighbor-joining tree nodes and branch lengths were statistically tested using a bootstrap method of 10,000 replicates and an interior branch test, respectively. The jModelTest program [59] was used to determine the most appropriate model for the analyses using the Akaike Information Criterion (AIC). Markov Chain Monte Carlo (MCMC) simulations were run for 5,000,000 generations with trees sampled every 1000 generations. Then Bayesian posterior probabilities were estimated after omitting the initial 1,000,000 generations. We sampled a tree every 100 generations and calculated a consensus topology for 7500 trees by omitting the first 2500 trees.

**Table 2:** Accession numbers of the control region of *B. gargarizans* mtDNA sequence in various regions of mainland China retrieved from NCBI GenBank.

	Locations	Latitude and longitude	Seq. No.	Accession No.
**a**	Yunnan, Jiangchuan	N24.27°,E102.73°	C3	DQ288705.1
**b**	Fujian, Wuyishan	N27.70°, E118.00°	C18	DQ288692.1
			C16	DQ288694.1
**c**	Fujian, Guadun	N27.43.89°, E117.39.36°	C19	AY924350.1
			C24	AF190234
**d**	Guizhou, Xishui	N28.33°,E106.20°	C31	DQ288709.1
**e**	Jiangxi, Xinjian	N28.70°,E115.80°	C13	DQ288695.1
			C17	DQ288693.1
**f**	Sichuan, Fushun	N29.2°,E105.0°	C33	AY924363.1
**g**	Hunan, Zhangjiajie	N29.3°, E110.4°	C22	AY924347.1
			C23	AY924346.1
**h**	Sichuan, E′mei	N29.60°,E103.40°	C25	DQ288715.1
**i**	Hubei, Shishou	N29.82°,E112.55°	C12	DQ288696.1
			C29	DQ288711.1
**j**	Chongqing, Nanchuan	N29.04.077°, E107.11.679°	C36	AY924351.1
**k**	Hunan, Shimen	N30.10°,E110.80°	C2	DQ288706.1
			C4	DQ288704.1
**l**	Zhejiang, Lin'an	N30.2°, E119.7°	C20	AY924349.1
			C21	AY924348.1
**m**	Anhui, Huangshan	N31.33°,E118.38°	C9	DQ288699.1
**n**	Sichuan, Wangyuan	N32.03.836°, E108.10.257°	C34	AY924362.1
**o**	Jiangsu, Yancheng	N33.38°,E120.13°	C1	DQ288707.1
			C11	DQ288697.1
			C8	DQ288700.1
			C7	DQ288701.1
**p**	Shanxi, Xi'an	N34.20°,E108.57°	C37	AF190235
**q**	Gansu, Lanzhou	N36.03°,E103.73°	C27	DQ288713.1
			C26	DQ288714.1
			C28	DQ288712.1
**r**	Shandong, Yiyuan	N36.18°,E118.17°	C5	DQ288703.1
			C6	DQ288702.1
			C10	DQ288698.1
**s**	Liaoning, Zhuanghe	N39.40°,E122.57°	C38	AF190233
**t**	Beijing, Baihuashan	N39.47.067°, E115.24.038°	C15	AY924364.1
			C32	AY924365.1
**u**	Tientsin, Jixian	N40.1°, E117.2°	C14	AY924367.1
**v**	Liaoning, Shenyang	N41.80°,E123.38°	C30	DQ288710.1
**w**	Heilongjiang, Suiyang	N45.75°, E126.63°	C35	AY924359.1

The second dataset was limited to the samples collected of this study. Partial sequences (564 bp) of 279 individuals of *B. bankorensis* from 27 populations in Taiwan and 4 individuals of *B. gargarizans* from Shanghai (mainland China) were analyzed. The numbers of haplotypes (N) and the values of haplotype diversity (*h*; [60]) and nucleotide diversity (π; [61]) were calculated using the DnaSP v5.0 software [62] (Table 1). The numbers of mutations between DNA haplotypes calculated in pairwise comparisons with MEGA v5.0 were employed to construct a minimum spanning network with the aid of MINSPNET [63]. Two measures of population differentiation, *G*_ST_ [64], which only considers haplotype frequencies, and *N*_ST_ [64], which considers similarities between haplotypes in addition to their frequencies, were compared to infer phylogeographic structure. A greater *N*_ST_ means that more closely related haplotypes occur in the same population, indicating the existence of phylogeographic structure at this scale [64]. The existence of phylogeographic structure was tested following Pons and Petit [64] by calculating two measures of genetic differentiation: *G*_ST_ and *N*_ST_. The global *N*_ST_ (0.792) was significantly higher than the global *G*_ST_ (0.312), providing evidence of the existence of phylogeographic structure because closely related haplotypes would be detected more frequently than that are less closely related in the same area [64]. The determination of *G*_ST_ and *N*_ST_ was carried out using DnaSP v5.0 [62].

#### Historical Demography

To analyze the population demographic history of *B. bankorensis*, Tajima's D statistic [65] and Fu's *Fs* test [66] of neutrality, the frequency distribution of pairwise differences between mtDNA haplotypes (mismatch distribution), and Bayesian skyline plots (BSP) were examined. The significances of Fu's *Fs* and Tajima's D values were evaluated using the coalescent algorithm in DnaSP v5.0 [62]. Evidence of population expansion within the lineages was obtained by determining mismatch distributions, implemented in DnaSP v5.0 [62]. This method is based on the premise that compared with a constant population size, population growth or decline leaves a distinctive signature in DNA sequences. A smooth and often unimodal pattern of the mismatch distribution (i.e., a large number of closely related haplotypes, indicating non-equilibrium conditions) reflects population expansion, whereas for stationary populations, the distribution is ragged and often multimodal (i.e., neutral, under equilibrium conditions) [67]. Furthermore, Harpending's raggedness index (Rg) [68] and the sum of squared deviations (SSD) were calculated using Arlequin v3.5 [69] to test whether the sequence data deviated significantly from the expectations of a population expansion model. Finally, for each BSP, the appropriate model of nucleotide substitution was determined using jModelTest. Genealogies and model parameters for each lineage were sampled every 1000th iteration for 20 million generations under a strict molecular clock with uniformly distributed priors and a pre burn-in of 2000. The effective sample size (ESS) for each of the Bayesian skyline analyses was greater than 200, suggesting that the 50 million generations were sufficient to estimate the demographic history for each lineage. This coalescent-based approach estimates the posterior distribution for the effective population size at intervals along a phylogeny, thereby allowing the inference of population fluctuations over time. These analyses were run for 1,000,000 generations, discarding the burn-in period. Plots for each analysis were drawn using Tracer v1.5 [70].

#### Population Genetic Differentiation

The program Arlequin v3.5 [69] was employed to estimate the *F*_ST_ values and their statistical significance between population pairs, i.e., the significance of population differentiation, with the following settings: 1000 permutations for significance and 10,000 steps in the Markov chain. When multiple comparisons were performed, the P values were adjusted using the sequential Bonferroni procedure [71]. A hierarchical analysis of molecular variance (AMOVA) was conducted using Arlequin v3.5 [69] to evaluate the most probable population configuration and geographic subdivision. The samples collected from different localities were grouped into 2 and 5 groups, according to the different geographic hierarchies that matched the geographic proximity. Samples collected from different localities were grouped together as follows to investigate the potential effects of various geographic barriers: (1) in two independent western and eastern groups (1–19; 20–27), which were primarily divided by the CMRs; and (2) in five groups (1–5; 6–12; 13–18; 19; 20–27) according to the major biogeographic zones in Taiwan based on the freshwater fish fauna [15] and other studies in frogs [18]. The Spatial Analysis of Molecular Variance (SAMOVA) 1.0 program [72] implements a simulated annealing procedure to define groups of geographically homogeneous populations that maximize the proportion of total genetic variance due to differences among population groups (*F*_CT_). These analyses were performed based on 1000 simulated annealing steps, and comparisons with the maximum indicators of differentiation (*F*_CT_ values) were conducted when the program was instructed to identify K  =  2–10 partitions for each sampling area within each analysis. The Mantel test [73] was performed using the computer program Alleles in Space (AIS) [74] to identify the correlations between the genetic and geographical distances among populations. Statistical significance was tested using 10,000 random permutations. Geographical distances were calculated based on the latitude and longitude of each population's collection location (Table 1). Finally, the genetic landscape shape of *B. bankorensis* was calculated using AIS. Using geographic data (projected in Universal Transverse Mercator) and the genetic distance scores calculated in AIS, a three-dimensional landscape, with genetic distance indicated by the z dimension was created. The output 3-dimensional values were projected onto a map of Taiwan Island to perform comparisons between the topographic and simulated genetic landscape.

Asymmetrical gene flow was observed among three regions: the western region, including localities 1–18; the eastern region, including localities 20–27; and the southern region, including locality 19. To estimate contemporary and historical gene flow among the three regions, maximum likelihood inference was performed using MIGRATE software, v 3.2.6 [75], [76]. The MIGRATE analyses were conducted using a full migration model (θ and M were estimated jointly from the data), which was compared with a restricted model (θ was averaged, and M was symmetrical between populations). The MIGRATE analyses were run five times to generate replicates using ten short chains (sampling 10,000 trees) and three long chains (sampling 100,000 trees), with a burn-in period of 10,000 trees, using an adaptive heating scheme. A migration matrix model with unequal population sizes and different migration rates was assumed [75].

#### Molecular Dating

The software BEAST v1.7.5 [77] was used to estimate the time to the most recent common ancestor (TMRCA) of the major mitochondrial lineages under an MCMC Bayesian approach by first dataset. Based on multiple fossil data and sequence comparisons, a divergence rate of approximately 3.50% per million years [36], [78] was estimated for the D-loop of *Bufo* mtDNA, and absolute TMRCA values were subsequently obtained [79], [80]. All analyses were performed using the HKY model of nucleotide substitution for the best-fitting model using jModelTest. Two independent Monte Carlo Markov chains were run for 100 million generations, with sampling every 10,000 generations and 10% burn-in of the posterior samples. The effective sampling size (ESS) parameter was found to exceed 200, which suggests acceptable mixing and sufficient sampling. The analysis was run five times to test the stability and convergence of the MCMC chains in plots of posterior log likelihoods in Tracer v1.5 [70]. The posterior samples from all runs were combined and analyzed in Tracer v1.5 to obtain mean estimates and the 95% highest posterior densities (HPD) of the TMRCA values.

## Results

### Phylogenetic Analysis

**Figure 2:**
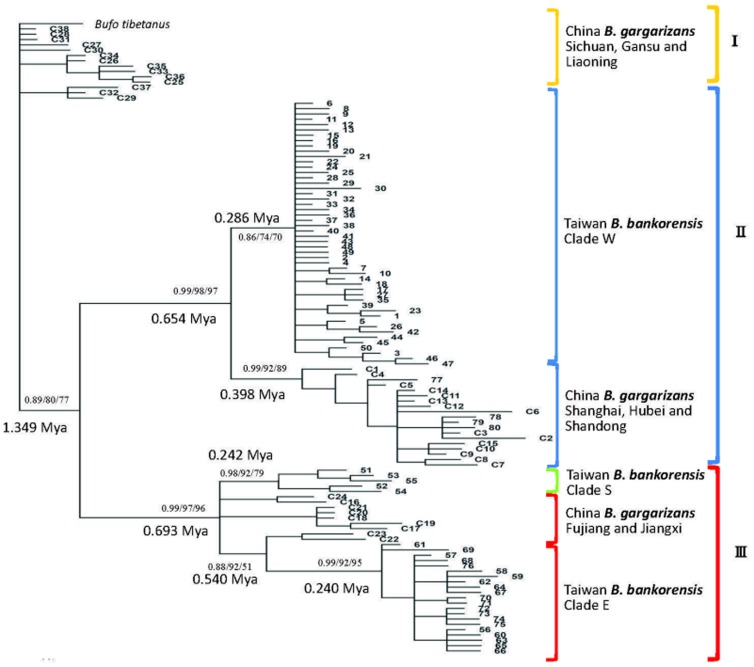
Phylogenetic trees reconstructed with MRBAYES from sequences of the mitochondrial D-loop gene in *B. bankorensis* and *B. gargarizans*. The values above the branches are the posterior probabilities for the Bayesian analysis and bootstrap values for the NJ and ML analyses. Bayesian phylogeny based on D-loop sequences (348 bp, n = 119) showing the relationships among Taiwan and mainland China phylogroups. Clade W, E and S were distributed in the western, eastern and southern CMRs in Taiwan Island, respectively.

**Figure 3:**
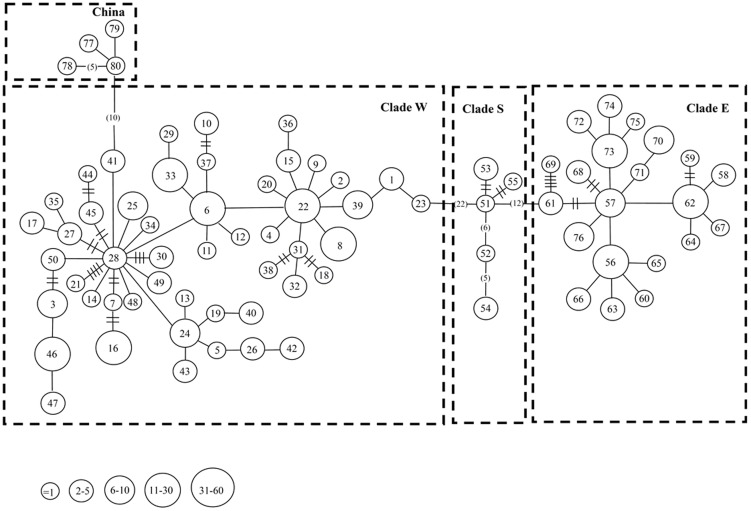
A minimum spanning network constructed using the 80 mitochondrial D-loop haplotypes identified in *B. bankorensis* and *B. gargarizans*. Haplotype designations (Table 1) are indicated next to each circle. Locality designations (see Figure 1) for specimens possessing each haplotype are indicated inside the circles. The sizes of the circles are proportional to the number of individuals represented. The length of the lines between circles is roughly proportional to the estimated number of mutational steps between the haplotypes.

For phylogenetic reconstruction, two approaches were used. Firstly, we combined 76 mtDNA D-loop haplotypes from 279 individuals in Taiwan and 4 haplotypes from Shanghai in mainland China of this study with 38 sequences of *B. gargarizans* in mainland China retrieved from NCBI GenBank (accession numbers listed in Table 2). This dataset had only 348 bp in alignment length. The optimum model of substitution selected by jModelTest was HKY+I for the D-loop gene. The topologies produced by ML, NJ and Bayesian analyses were highly similar, and branch support values were generally similar or identical, thus only the Bayesian trees are shown (Figure 2). Phylogenetic analyses of the dataset with a length of 348 bp recovered a closed relationship between *B. bankorensis* and *B. gargarizans* (Figure 2). Three distinct lineages were identified: (1) Lineage I, including *B. gargarizans* from the Sichuan, Gansu and Liaoning in mainland China; (2) Lineage II, including *B. gargarizans* from the Shanghai, Hubei, and Shandong in mainland China and *B. bankorensis* form Clade W in Taiwan; (3) Lineage III, including *B. gargarizans* from the Fujiang and Jiangxi in mainland China and *B. bankorensis* form Clade E and S in Taiwan. *B. gargarizans* from mainland China were split into two lineages (II & III) approx. 1.349 million years apart. *B. bankorensis* was also found in these two lineages. The second dataset was limited to the samples collected for this study. The mtDNA D-loop (564 bp length) of 279 individuals of *B. bankorensis* from 27 localities in Taiwan and 4 individuals of *B. gargarizans* from Shanghai (mainland China) were analyzed. The alignment of all D-loop sequences revealed 80 unique haplotypes and the minimum-spanning network was reconstructed based on mutational changes (Figure 3). The network showed that 4 main clades were congruent with the phylogenetic reconstruction and showed great genetic differentiation (22 mutational steps between Clade W and Clade S; 12 mutational steps between Clade E and Clade S; 10 mutational steps between Clade W and population from Shanghai, Figure 3). Additionally, Clade W and Clade E were widespread in the western and eastern CMRs, respectively. While Clade S only distributed in southern Taiwan (Figure 3). The divergence between Clade E and Clade S was lower than that of Clade E and Clade W. The average genetic distance between the clades corresponded to 5.0%, which was approximately six-fold greater than that within the clades (averages of 0.7 and 0.8% for Clade W and Clade E plus Clade S, respectively). Clade W contained a total of 51 haplotypes from 186 samples, Clade E consisted of 21 haplotypes from 86 samples, and Clade S was 5 haplotypes from 7 samples (Table 3). Most haplotypes of *B. bankorensis* found in the western and eastern sides of the CMRs were unique accordingly to geography. Only four haplotypes shared across the CMRs occurred at localities 26 and 27, which included haplotypes from two highly divergent lineages (Clade W and Clade E) (Table 1).

**Table 3:** Summary of the samples sizes, haplotype numbers, haplotype diversities (*h*), nucleotide diversities (π), Tajima's D and Fu's (*Fs*) tests, Ramos-Onsins and Rozas' R_2_ and mismatch estimation results, including SSD and Rg values, for D-loop region sequences in the total population and each clade in Taiwan Island.

Lineage	Sample size	Haplotype numbers	Haplotype diversity (*h*)	Nucleotide diversity (π)	Tajima's *D*	Fu's *F_S_*	R_2_	SSD	Rg
**Total**	279	76	0.973	0.026	0.216	−16.448***	0.095	0.053	0.019
**Clade W**	186	51	0.958	0.007	−1.972*	−39.603***	0.039	0.001	0.020
**Clade S**	7	5	0.905	0.009	0.672	0.313	0.202	0.082	0.222
**Clade E**	86	21	0.909	0.004	−1.658	−11.023***	0.046	0.017*	0.079***

*P < 0.05, ***P < 0.001.

### Genetic Diversity of *B. bankorensis*

The sample size, number of haplotypes, and values of nucleotide diversity (π) and haplotype diversity (*h*) within each population are presented in Table 1. Overall, the mean haplotype diversity (*h*) among the 279 samples was estimated to be 0.973, and the mean nucleotide diversity (π) was 0.026 (Table 1). Haplotype diversity ranged from 0.000 to 1.000, and the nucleotide diversity (π) within populations varied from 0.000 to 0.032 (Table 1). The localities showing the highest nucleotide diversities in ZJ, CM, and SK (localities 15, 26, and 27 with diversities of 0.016, 0.032, and 0.014, respectively) resulted that individuals from two different clades, eg. Clades W and E. The most common haplotype (hap 6) was found in 23 individuals across 8 localities in the western populations. Among the eastern populations, the most common haplotype (hap 56) was found in 14 individuals across 3 localities. The geographical distribution of the mtDNA haplotype frequency is illustrated in Figure 1B.

### Population Genetic Structure

The genetic variation of *B. bankorensis* was highly structured, as indicated by significant *F*_ST_ values and genealogical estimates. Some of the mitochondrial haplotypes were geographically widespread, but most of the shared haplotypes occurred between closely related populations in geographically proximate populations, indicating current gene flow or expansion over a long distance. Pairwise *F*_ST_ tests indicated significant genetic differentiation among the localities (−0.126 to 1.000). Most of the pairwise *F*_ST_ values were significant (*P* < 0.05). The overall standardized *F*_ST_ value among all samples was 0.790. In the hierarchical analysis, all localities were separated into two groups and tested via AMOVA. In the first group was tested CMRs geographic division, most of the molecular variance (80.10%) was corresponded to variation between groups, while 12.50% was attributed to variation within populations, and 7.40% of the molecular variance was related to variation among populations within groups. In the second group was tested five biogeographic zones, most of the variation was attributed to between group variation (75.37%), while 18.36% corresponded to the variation within populations, and 6.27% of the molecular variance was related to the variation among populations within groups (Table 4). The spatial analysis of molecular variance (SAMOVA) indicated that the *F*_CT_ value was the highest for K  =  3 (*F*_CT_  =  0.825, P  =  0.000): KT (locality 19) was assigned to one group, and the remaining populations formed two groups, corresponding to the western region (localities 1–18) and the eastern region (localities 20–27). Most of the remaining variation was found within populations (12.57%) or among populations within groups (4.88%). All of the variance components were significant (P  =  0.000). The Mantel test (r  =  0.0448, P  =  0.074) was detected a very weak correlation between genetic distances and geographic distances. This result indicated that localities within the same region are comparatively homogenous and that localities in different regions present high genetic differentiation. An analysis of the shape of the genetic landscape for *B. bankorensis* was calculated using AIS [74] and projected onto a map of Taiwan (Figure 1). This simulated landscape (Figure 1C) exhibited an almost identical shape as compared to the actual topographic landscape (Figure 1B), with CMRs separating the island into the eastern and western regions.

**Table 4:** AMOVA results testing the genetic subdivision between populations based on mtDNA sequences among geographic districts of Taiwan Island.

	Sum of squares	Percentage of variation	Fixation indices	Significance tests
**Groups: (K = 2)**				
Among groups	1375.723	80.10	Φ_CT_ = 0.801	P = 0.000
Among populations within groups	300.286	7.40	Φ_SC_ = 0.371	P = 0.000
Within populations	435.615	12.50	Φ_ST_ = 0.875	P = 0.000
**Groups: (K = 5)**				
Among groups	1505.967	75.37	Φ_CT_ = 0.753	P = 0.001
Among populations within groups	170.042	6.27	Φ_SC_ = 0.254	P = 0.000
Within populations	435.615	18.36	Φ_ST_ = 0.816	P = 0.000

The estimates of historical migration rates (M) calculated using MIGRATE indicated asymmetrical gene flow among regions over the long term at both sides of CMRs. The results revealed that the degree of historical gene flow from the western region to the eastern and southern regions was greater (M_W→E_  =  106.63, M_W→S_  =  87.87) than it was in the opposite direction (M_E→W_  =  12.24, M_S→W_  =  12.58). Furthermore, the similar asymmetrical direction and strength of individual migration toward the south was also detected in southeast Taiwan (M_E→S_  =  163.18, M_S→E_  =  53.84). Accordingly, the mtDNA evidence, characterized by a distinct inheritance mode, indicated that the western region acts as a source, while the populations of the eastern and southern regions act as a sink (Table 5).

**Table 5:** Summary of *B. bankorensis* immigration rates among the western, eastern, and southern regions estimated using MIGRATE.

	MIGRATE Region			
**Region**	**θ**	**Western**	**Eastern**	**Southern**
**Western**	0.0284	–	106.63	87.879
**Eastern**	0.0138	12.24	–	163.18
**Southern**	0.0051	12.58	53.84	–

### Demographic History

**Figure 4:**
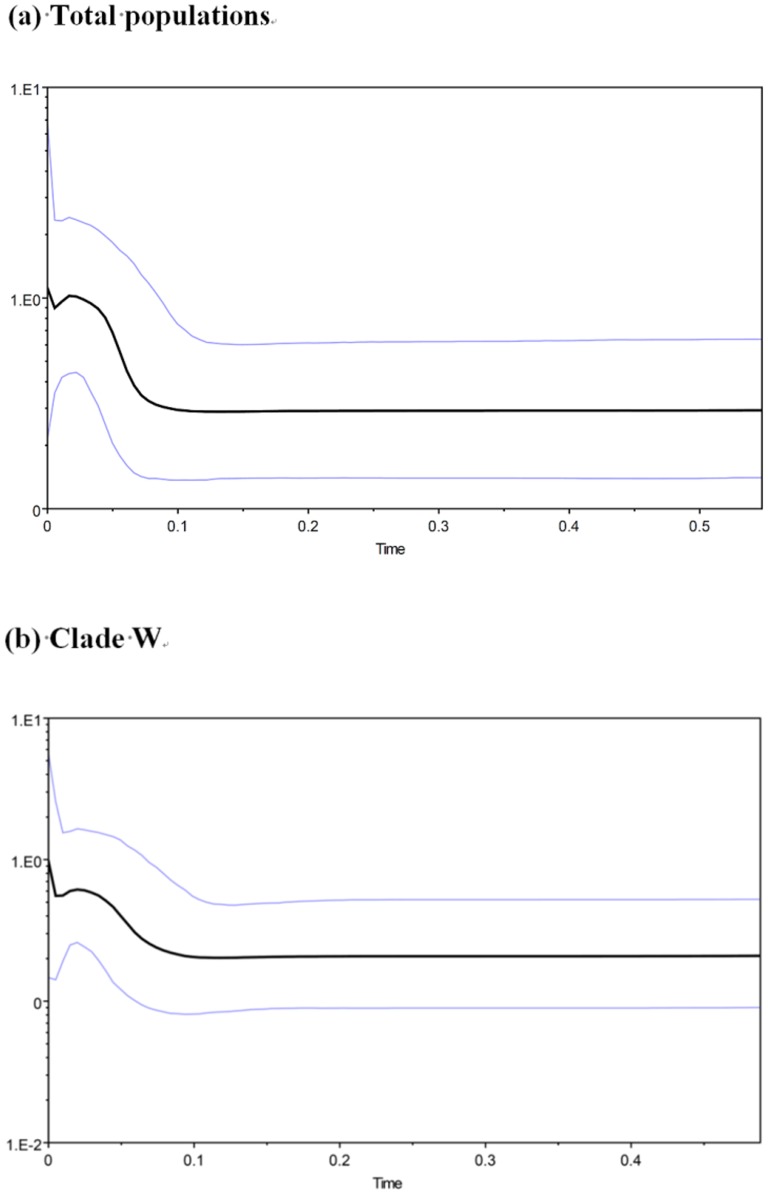
Bayesian skyline plot of the effective population sizes over time for *B. bankorensis*. The X axis is the time before the present in units of million years ago, and the Y axis is the estimated effective population size in units of Neτ, i.e., the product of the effective population size and the generation length in years (log transformed). (a) Total populations and (b) Clade W.

An examination of demographic histories revealed marked differences between clades and total populations in this study. The sequence variation (Table 3) observed in each clade was similar to our estimated *h* and π values. A significant negative deviation from the neutrality test could reflect past population expansion events. The mismatch analysis generated an unimodal curve showing non-significant SSD and Rg index values (*P*> 0 .05, Table 3), suggesting significant population expansion for the total population and Clade W. However, Tajima's D value for the total population was positive (0.216) and statistically non-significant. Fu's *Fs* test has been shown to be much more sensitive in detecting population growth than Tajima's test [66]. Based on the estimated divergence rate of approximately 3.50% per million years [36], [78], the expansion time for Clade W and total population were estimated approximately 0.07 and 0.08 Mya, respectively (Figure 4).

### Molecular Dating

The evolutionary rate within *B. bankorensis* was calculated to be 3.50% per million years [36], [78] via MCMC simulation. A strict clock model was implemented in BEAST to estimate the TMRCA values of the different clades and the time since clade separation [77]. The analyses using BEAST indicated that the TMRCA for Lineage II and Lineage III dated to 1.349 Mya (effective sampling size, ESS = 5892.001, 95% credibility interval 0.964–1.737 Mya). Molecular dating estimated that the Lineage II and Lineage III coalesced to their TMRCA values of 0.654 ± 0.279 and 0.540 ± 0.285 Mya, respectively. The TMRCA values for Clades W, E, and S were dated to 0.286 ± 0.150, 0.240 ± 0.163, and 0.242 ± 0.159 Mya, respectively. This result suggests that the molecular dating of *B. bankorensis* was tightly coupled with the formation of the CMRs after the Pleistocene epoch.

## Discussion

### Genetic Variation within *B. bankorensis*

The evolution of a recently established population on an island is affected by the founder event itself, but genetic drift shapes the diversity and the divergence of island populations over time: island species generally present lower genetic variability and higher differentiation among populations than do closely related species on the mainland [81], [82]. Our results revealed lower genetic variability in *B. bankorensis* (mean π =  0.026) than has been found in other studies on *Bufo* species from mainland China based on D-loop sequences, e.g., *B. gargarizans* (mean π =  0.047) [79]. The existence of a high *h* and relatively high π value (0.973 and 0.026) in the total population appears to reflect the mixing of differentiated clades analyzed together [2], [83], [84]. Furthermore, the *h* and π values (0.960 and 0.009, respectively) obtained for the western region (localities 1–18) were similar to those for the eastern region (localities 20–27) (0.919 and 0.009) (Table 1). Concerning the pattern of nucleotide diversity among *B. bankorensis* populations, the western region showed a similar π value to the eastern region, which is contrary to previous studies in *B. robusta* [20] and *S. latouchii* [22] showing higher π value in the western region of the CMRs. The colonization ability of *B. bankorensis* at higher altitudes may broaden its distribution range, thus enabling the east to harbor more genetic variation due to a larger population size than is observed in other frog species [85]. The higher nucleotide diversities observed at three localities (ZJ, CM, and SK) were due to the co-existence of individuals from two clades (W&S; W&E). Furthermore, the similar haplotype compositions observed in populations on both sides of the CMRs indicate a dispersal mechanism possibly associated with secondary contact or human activity. However, migration along traffic routes or via unintended carriers does not appear to be dominant. The involvement of refugia or secondary contact seems most likely; though verifying the roles of these phenomena require further study.

### Phylogeography of *B. bankorensis*

Phylogeographic and landscape genetic analyses provide insight into the history of populations across their geographic range at different spatial scales. Two monophyletic lineages were obtained in our phylogeographic analysis: lineage II and lineage III (Figure 2). In the linage II, the clade W of *B. bankorensis* formed a monophyletic clade with *B. gargarizans* samples from northern China (Shanghai, Hebei, and Shandong), while the clades E and S of *B. bankorensis* in the linage III clustered together with *B. gargarizans* samples from southern China (Fujiang and Jiangxi) composed another monophyletic lineage. Accordingly, this result suggested a colonization history of *B. bankorensis* in Taiwan that could be explained by two distinct origins: populations on the west side of CMRs originated from northern China, while populations on the east side of CMRs initiated from southern China (Figure 1A, 1B), respectively. Our observations of *B. bankorensis* revealed significant levels of genetic structure across its distribution in Taiwan (Figure 1B, 1C and Figure 3). All of our analyses indicated the presence of two genetically well-differentiated clades (Clade W and Clade E plus Clade S) of *B. bankorensis* within Taiwan. This implies that the CMRs serve as a genetic barrier to *B. bankorensis* and separated the whole island into the eastern and western sides during its formation. A general pattern of E-W deviation is consistent with previous phylogeographic studies in other taxa on Taiwan Island, such as freshwater fishes (*V. barbatulus* [18]; *A. kikuchii* [19]), frogs (*Fejervarya limnocharis* [86]; *B. robusta* [20]; *S. latouchii* [22]), reptiles (*Trimeresurus stejnegeri* [87]; *Naja atra* [88]), and mammals (*Callosciurus erythraeus* [89]).

Three biogeographical scenarios can be suggested according to the obtained phylogenetic trees and network (Figure 2 and Figure 3). Clade E occurs in the eastern region of the CMRs along the coast of the Pacific Ocean, while individuals collected from the south of Taiwan constitute Clade S (Figure 1). Some individuals from Clade S coexisted in the members of Clade W, though Clade S distribution was largely restricted to the southern part of the Kaoping River. The Kaoping River is the largest river drainage system in southern Taiwan and may act as an effective geographical barrier for isolating populations of *B. bankorensis*. In addition, the southern region harbors the distinct *B. bankorensis* haplotypes, which has also been observed in freshwater crabs (*Candidiopotamon rathbunae* [90]), freshwater fishes (*Candidia barbatus* [91]), and frogs (*Sylvirana latouchii* [22]). This pattern of phylogeographic outliers suggests that these populations show a history of isolation from the populations in southern Taiwan and present a relatively high conservation value. In previous studies, two topological barriers, the Miaoli Plateau and Formosa Bank, have been found to represent significant barriers to gene flow in species from various taxa in western Taiwan, such as bagrid catfishes [92], cyprinids [18], [23], [93], frogs [20], [22], [94], snakes [87], [88], and squirrels [89]. Despite the previously reported evidence of amphibian species divergence being caused by a combination of topological barriers and river systems, as for *B. robusta* [20], such effects are not detected in *B. bankorensis* populations. Two mechanisms are postulated for the neutral effects of topological barriers on *B. bankorensis*. Firstly, male *B. bankorensis* are able to mate with females in river streams as well as temporary ponds during the breeding season. Secondly, *B. bankorensis* shows a broad distribution at various altitudes and presents excellent mobility over long distances.

### Genetic Structure and Differentiation in *B. bankorensis*

The average *F*_ST_ value of the overall population was 0.790 (*P*<0.05), which indicates a high degree of genetic differentiation among populations of *B. bankorensis*. The genetic differentiation among *B. bankorensis* populations showed a weak pattern of isolation by distance (Mantel test, r  =  0.0448, P  =  0.074), as expected for a toad separated by the CMRs, revealing that the isolation by distance pattern might not be the driving force for the differentiation of the identified clades. Although isolation by distance is typically considered to be a consequence of restricted contemporary gene flow, differentiation over distance can also result in an interplay between modern and vicariant forces, because particularly historical events are more likely to be detected at larger geographical scales [95]. The phylogenetic trees (Figure 2) showed that the W, E and S clades of *B. bankorensis* are nested with *B. gargarizans* lineages from mainland China. Furthermore, the fact that Clades E and W of *B. bankorensis* are not closely related indicates that they represent distinct evolutionary origins. The divergent genetic clades were found within *B. bankorensis* thus represent species polyphyly as a result of independent colonizations by previously diverged lineages of *B. gargarizans* complex. This is consistent with our recent study of genetic structure of *B. bankorensis* cyt *b* [96], which concluded that one linage (western group 1, uncut by *Bam*HI and cut by *Tsp*RI) is most likely *B. gargarizans*, a second one (western group 2, uncut by both *Bam*HI and *Tsp*RI) is *B. bankorensis*, and a third one (eastern clade, cut by *Bam*HI but not cut by *Tsp*RI) may be a new subspecies. *B. bankorensis* has been historically recognized as one endemic species [28], has distinct morphological characters as compared with *B. gargarizans* and is restricted to Taiwan, however the molecular evolutionary relationships fail to support this taxonomy. Hence, the taxonomic status of *B. bankorensis* and *B. gargarizans* is still unclear and needs further exploration.

The results of AMOVA and SAMOVA revealed that the mtDNA D-loop dataset from *B. bankorensis* can be best partitioned into three groups. These three groups correspond to topological barriers plus the major mtDNA clades identified in *B. bankorensis*. A large proportion (82.5%) of the genetic variability found in *B. bankorensis* can be explained by variance among groups (Table 4). This can primarily be attributed to the deep divergence observed among clades. The high genetic variability among populations is also evident in the many significant pairwise *F*_ST_ values obtained, suggesting a low level of gene flow between populations, even among clades. Asymmetrical gene flow was observed among three regions. Kaouping River was used to be significant a landscape barrier for species in the southern Taiwan, i.e. *Zacco pachycephalus* [93], and *Candidia barbata* [91], where the north-south gene flow would be blocked and then lead to population diversification. In this study, the populations 15, 16, 17, and 18, located in the Kaouping River region, should geographically share closer genetic proximity to population 19 than others in the western Taiwan. However, the anthropogenic activities, such as developed traffic network, would contribute to counteract the effect of geographical barrier and promote the reconnection among populations. Thus, except for population 19 at the extreme south, the west region including populations from 15 to 18, was genetically separated from the southern population due to homogenizing effect on the W clade. In addition, the asymmetrical individual migration toward the south would be associated closely with the relatively smaller population size in S clade, which was more vulnerable to population disturbance than the vice versa.

The TMRCA analysis traced the divergent time between lineage II and lineage III back to approximately the Pleistocene (1.349 Mya), which is similar to the previous results of *B. robusta* [20]. Given such a distant time of divergence and the fact that the rise of the CMRs occurs approximately 2.50–1.00 Mya [97], [98], the populations appear to exhibit largely independent histories with limited gene flow. The estimated TMRCA for Clade E and Clade S is more recent than that of Clade W, which has also been observed for *B. robusta* [20]. We conclude that the recent migration from the western region toward the eastern sink region might be largely responsible for such a scenario. Nevertheless, the gene flow between Clade W and Clade E plus Clade S (i.e., haplotype sharing among localities) was found to be extremely low, revealing either gene flow or the retention of ancestral polymorphisms. These historic complexities in the landscape have likely played important roles in the distribution of habitat availability and possibly even resulted in the later recolonization of the eastern and southern locations from the western region as the area of suitable habitat expanded during glacial recessions in the Pleistocene. This scenario implies that migration was asymmetric, and the western region is suggested to be the most important contributor to gene flow. In some cases, high migration rates were suggested, particularly from the eastern region to the southern region. However, secondary contact may have occurred with the increasing amount of shoreline along the Hengchung peninsula during the Middle Pleistocene [14], which facilitated communication between the southern and eastern populations (e.g., in the *Loxoblemmus appendicularis* complex [99]).

### Historical Population Demography

Nucleotide and haplotype diversities can provide information on evolutionary histories. Our results revealed high haplotype diversity and low nucleotide diversity in the tested *B. bankorensis* populations, which is a pattern that could occur following population expansion [84] (Table 3). In the Pleistocene, glacial advances transformed the physical and biological environments of southern Asia [100]. The climatic changes accompanying with the beginning of glaciation drove high-latitude populations into more southern habitats in the Northern Hemisphere [101], [102]. In Taiwan, the contraction-expansion model predicts that populations are affected by these habitat shifts underwent rapid population expansion as previously unsuitable habitat became available for colonization. Despite differences in geography, rapid or step-wise colonizations would be characterized by low levels of genetic diversity, as each new founder population represents only a fraction of the ancestral population's genetic diversity [10], [103]. Our demographic analyses show that the intra-clade genetic structure of *B. bankorensis* contains signatures of demographic expansion consistent with Pleistocene glacial retreat. Specifically, the demographic and genetic variation analyses of total populations and Clade W provide a strong support for the existence of recent population expansions, represented by significantly negative *Fs* values and unimodal mismatch distributions with a low Rg index (Table 3), and the BSPs is consistent with the estimated times of these expansions range from 0.07 to 0.08 Mya (Figure 4). In contrast, no population expansion was detected in Clade E and Clade S, which is inconsistent with the previous results for the other frog species, e.g., *B. robusta* [20] and *S. latouchii* [22], demonstrating historical population expansion in the same region. The eastern Taiwan populations of *B. bankorensis* presented a smaller scaled effective population size (θ) than populations in western Taiwan (Table 5), which is consistent with a history of spatial expansion. Based on the broad distribution of *B. bankorensis*, ranging from the sea level to above 3,000 m in altitude, population expansion is expected to have occurred according to habitat release during the glaciated period, especially in the western Taiwan is associated with a significant decline of the sea level. In contrast, the eastern Taiwan is surrounded by the Mariana Trench, where a dramatic decline in depth would further constrain the rapid outward colonization of *B. bankorensis*, hence greatly reducing the probability of typical population dynamics occurring during glacial-interglacial periods. The CMRs of Taiwan approach to an elevation of nearly 4,000 m, rising steeply from the eastern coast and giving way to a broad western plain. The land mass on the eastern side of Taiwan, which faces the margin of the Pacific Ocean, was not considerably altered during glacial–interglacial periods, resulting in limited range expansion for the eastern clade. In contrast, the decrease in sea level on the western side of the island caused the Taiwan Strait to dry up. Consequently, the habitat of *B. bankorensis* was reconnected with the mainland China, giving rise to new ecosystem through gene flow by means of re-migration and/or population expansion.
